# Changes in Traumatic Memories and Posttraumatic Cognitions Associate with PTSD Symptom Improvement in Treatment of Multiply Traumatized Children and Adolescents

**DOI:** 10.1007/s40653-019-00255-3

**Published:** 2019-04-04

**Authors:** Samuli Kangaslampi, Kirsi Peltonen

**Affiliations:** Faculty of Social Sciences / Psychology, Tampere University, FI-33014 Tampere, Finland

**Keywords:** PTSD, Intervention, Mechanism, Cognition, Memory, Children, War

## Abstract

Refinement, targeting, and better dissemination of trauma-focused therapies requires understanding their underlying mechanisms of change. Research on such mechanisms among multiply traumatized children and adolescents is scarce. We examined the role of improvements in problematic qualities of traumatic memories and maladaptive posttraumatic cognitions in PTSD symptom reduction, in a randomized, pragmatic trial of narrative exposure therapy vs. treatment as usual with 40 participants 9–17 years old (48% female, 75% refugee background) repeatedly exposed to war or family violence related trauma. Posttraumatic cognitions, quality of traumatic memories and PTSD symptoms were assessed by self-report before and after treatment. Improvements in both quality of traumatic memories (*r*_MI_ = .36) and posttraumatic cognitions (*r*_MI_ = .46) correlated with symptom reduction. However, improvement during treatment was only significant for quality of traumatic memories (*F*_MI_(11,333.56) = 4.77), not for posttraumatic cognitions. We detected no difference in effects of narrative exposure therapy and treatment as usual on cognitions or memories. We tentatively suggest problematic, overly sensory and incoherent quality of traumatic memories may be a useful target in the treatment of PTSD symptoms among multiply traumatized children and adolescents. Changing maladaptive posttraumatic cognitions, though important, may be challenging among those with severe, repeated trauma.

Meta-analyses have found several trauma-focused treatments effective in treating posttraumatic stress symptoms (PTSS) among children and adolescents (Brown et al. 2017; Gillies et al. 2016). However, it is largely unclear to what extent they achieve their effects via the same or dissimilar processes. Identifying both shared and unique mechanisms of change that interventions tap into for their effectiveness is crucial for further development, effective dissemination and improved targeting of treatment approaches (Ehlers et al. 2010; Kazdin 2007; Zalta 2015). Simultaneously, regardless of type of treatment, assessing the relevance for recovery of particular mechanisms suggested as central by different theories of posttraumatic stress disorder (PTSD) provides for good tests of each theory’s predictions. Here, we study mechanisms of change potentially involved in the treatment of PTSS among multiply traumatized children and adolescents.

This study is a preregistered, secondary analysis of data from a randomized, controlled trial comparing narrative exposure therapy (NET; Schauer et al. 2011) with treatment as usual (TAU), in a usual care, clinical setting (for details, see Peltonen and Kangaslampi 2019). Narrative exposure therapy is a manualized, short-term, trauma-focused cognitive-behavioral therapeutic intervention for adults and children who suffer from PTSS due to repeated traumatic events. NET differs from other exposure-based treatments by its emphasis on narrative reconstruction of the trauma survivor’s autobiography by chronologically visiting and exposing the client to each traumatic memory in detail. As such, the NET protocol considers reintegration and contextualization of traumatic memories as central to treating PTSS (Schauer et al. 2011; Mørkved et al. 2014). NET was developed, and has mainly been studied, in the context of traumatic experiences related to war or armed conflict, with substantial evidence for effectiveness (reviewed in Robjant and Fazel 2010; Mørkved et al. 2014). Among adults, NET has also been successfully trialed with Saudi firemen with work-related trauma (Alghamdi et al. 2015), Chinese earthquake survivors (Zang et al. 2013) and women with comorbid borderline personality disorder and PTSD due to various trauma (Pabst et al. 2014). Four randomized controlled studies have previously demonstrated the effectiveness of NET with children or adolescents, all with conflict-related trauma (Catani et al. 2009; Ertl et al. 2011; Ruf et al. 2010; Schaal et al. 2009). The present study included both children and adolescents traumatized by experiences of war, armed conflict and refugeedom, as well as those who had experienced multiple traumatic events related to domestic physical or sexual abuse.

Despite increasing evidence for effectiveness in a variety of contexts, no studies have so far examined mechanisms of change involved in symptom improvement during NET treatment, neither on its own nor compared with other active treatment. Here we focus on two putative mechanisms: changes in maladaptive posttraumatic cognitions and improvement in the quality of traumatic memories.

Posttraumatic cognitions (PTCs) refer to dysfunctional or maladaptive appraisals of the traumatic event and its consequences. These overly negative, generalized or catastrophizing appraisals and beliefs typically relate to the self as fragile, incompetent or permanently changed for the worse due to the trauma or to the world as dangerous, scary, and unpredictable (Foa and Rothbaum 1998; Meiser-Stedman et al. 2009). Information processing accounts of PTSD suggest maladaptive PTCs have a key role in preventing recovery from PTSS by keeping up a sense of on-going threat and promoting the use of dysfunctional strategies to reduce distress (Ehlers and Clark 2000; Dalgleish 2004). Thus, they would be important targets for treatment, which developers of evidence-based PTSD treatments have also acknowledged (Schnyder et al. 2015).

The evidence base for the role of improvements in maladaptive PTCs in PTSS treatment by psychological methods is already quite robust among adults (Sripada et al. 2016; Zalta 2015). Change in maladaptive PTCs appears a rather general mechanism that many types of treatments might draw upon. Fine-grained longitudinal analyses suggest that it is indeed change in such appraisals and beliefs that leads to reduction in symptoms, and not vice versa (Kumpula et al. 2017; Kleim et al. 2013) – a crucial condition for claiming a mechanistic role (Johansson and Høglend 2007). Among children and adolescents, some previous research has identified change in maladaptive PTCs as a mechanism of change for decreased PTSS in cognitive (Meiser-Stedman et al. 2017; Smith et al. 2007) and exposure-based treatments (McLean et al. 2015). Yet, none of these studies has included children with trauma related to war or refugeedom.

Research suggests that exposure-based treatments like prolonged exposure have their effects partly via cognitive change (Cooper et al. 2017; Kumpula et al. 2017), so we might expect this to be the case for NET, as well. Despite the lack of overt cognitive restructuring, appraisals of the traumatic event and its meaning may change when aspects of the trauma are recollected or reconstructed that contradict or disconfirm maladaptive beliefs (Ehlers and Clark 2000; Foa and Rothbaum 1998; Schnyder et al. 2015) and the meaning of the traumatic event is reflected upon after exposure (Schauer et al. 2011). Alternatively, through integration of traumatic memories into a coherent life narrative, trauma-related thoughts and emotions might become better linked to their specific circumstances and context, correcting or attenuating over-general appraisals of ever-present threat (Schauer et al. 2011).

Several theoretical accounts of PTSD also suggest the problematic dual nature of traumatic memories to be intimately linked to PTSS (Brewin 2014; Ehlers and Clark 2000; Foa and Rothbaum 1998). On one hand, excessively sensory-based, non-declarative traumatic memories are easily recalled involuntarily based on environmental and internal cues, leading to distressing intrusive memories or flashbacks. While verbally and voluntarily accessible episodic memories of the trauma are suggested to be fragmented, disorganized, and/or poorly spatiotemporally contextualized in individuals suffering from PTSD. Despite somewhat differing terminology and emphasis, developers of evidence-based PTSD treatments have agreed on reorganization of memory as an important goal for trauma-focused treatment (Schnyder et al. 2015).

Previous research has examined trauma memory characteristics and their links to symptoms either by analyzing trauma narratives provided by survivors or by using self-report instruments of the qualities of traumatic memories. In the first tradition, evidence on whether memories of traumatic events are more fragmentary or less cohesive among those with significant PTSS is mixed (Brewin 2014; O'Kearney et al. 2007; Rubin et al. 2016; Salmond et al. 2011). Further, fragmentation in trauma narratives does not necessarily decrease with successful treatment, casting further doubt on the specific relevance of narrative fragmentation for PTSS (Bedard-Gilligan et al. 2017; Desrochers et al. 2016). The present study focused on the self-reported quality of traumatic memories, which has also been characterized as perception of memory quality (McKinnon et al. 2017). Previous studies using self-report measures likewise provide mixed results. Following adult survivors of recent assault over six months, Halligan et al. (2003) found those with current PTSD to report more disorganized traumatic memories. The self-reported disorganized quality of the traumatic memory was associated with PTSS up to six months later. However, change in self-reported disorganization was not related to changes in PTSS severity in this study. Among children, Salmond et al. (2011) did not find self-reported problematic qualities of traumatic memory to predict acute stress symptoms beyond the effect of narrative disorganization. McKinnon et al. (2017) reported that self-reported memory quality was a stronger cross-sectional and prospective predictor of PTSS than features of the trauma narrative. In that study, change in self-reported trauma memory quality was also linked to change in PTSS over three months after the trauma. All these studies examined naturalistic change after single-incident trauma. To our knowledge, no similar studies exist among multiply traumatized children or adolescents, representing a significant gap in knowledge.

McKinnon et al. (2017) called for their results to be replicated in the context of change due to therapy. So far, just one study has specifically looked at improvements in the quality of traumatic memories, whether as narratives or self-reports, as a mechanism of change in treatment of PTSS. In a study on cognitive therapy among children and adolescents suffering from PTSD, again due to recent single-incident trauma, Meiser-Stedman et al. (2017) found that improvements in the self-reported quality of traumatic memories over the course of the treatment were linked to the treatment’s effects on PTSS. However, when changes in memories from pre-intervention to just midway through it were examined instead, they were not found to significantly mediate pre-post improvements in symptoms. Thus, the authors were unable to demonstrate the temporal sequence of changes in traumatic memories and PTSS.

In light of this disparity between major theoretical emphasis, mixed indirect evidence and almost total lack of direct evidence for a mediating role, research on changes in the quality of traumatic memories during PTSD treatment is sorely needed. As integration and contextualization of traumatic memories is a central aim and focus in its protocol, NET might be especially suited for exploring this putative mechanism. At the same time, examining whether improvements in the quality of traumatic memories can also occur during mainly non-trauma-focused TAU provides an important point of comparison.

The present study has two main objectives. First, we examine changes in maladaptive posttraumatic cognitions and in self-reported problematic qualities of traumatic memories during treatment of posttraumatic stress symptoms among multiply traumatized children and adolescents in a usual care environment, and possible differences between narrative exposure therapy (NET) and treatment as usual (TAU) in these changes. We also explore whether such changes in cognitions and memories are related to reduction in posttraumatic stress symptoms. We hypothesize that, while both maladaptive posttraumatic cognitions and problematic qualities of trauma memory representations are likely to decrease during treatment for both groups, these decreases are greater in NET than during TAU. Second, we aim to test whether changes in posttraumatic cognitions and the quality of traumatic memories act as mechanisms of change responsible for NET’s effectiveness in reducing posttraumatic stress symptoms, compared with TAU.

## Method

### Participants

The participants of this study were children and adolescents 9–17 years of age receiving treatment at cooperating units for stress symptoms evaluated to result from exposure to repeated trauma. Exclusion criteria included acute psychosis, active suicidal ideation, active serious substance abuse, and intellectual disability.

Due to the pragmatic nature of the trial, with interventions and assessments taking place at a variety of different usual care environments, there were some dropouts (eight out of 50 before end of treatment) and a substantial share of data was missing. This analysis concerns those children and adolescents who completed NET or TAU and for whom at least some measurements after treatment were available, a final sample of 40 participants. Reasons for dropout are reported elsewhere (Peltonen and Kangaslampi 2019).

The participants of this study were 9–17 years old (*M* = 13.30; *SD* = 3.06), with 19 girls and 21 boys. Ten participants were born in Finland and had experiences of family violence, while 30 participants were born outside Finland, most commonly in Iraq (*n* = 11) or Afghanistan (n = 11), and had experiences of war or refugeedom. Of this sample, 23 received NET, while 17 completed TAU.

### Procedure

This analysis is based on data from a randomized, controlled, open-label, multisite, pragmatic trial of NET vs. TAU in usual care environments. The trial was registered on ClinicalTrials.gov (NCT02425280) before any work on it commenced. The study protocol of the trial was also published beforehand (Kangaslampi et al. 2015). Plans for analysis of mechanisms of change were included in this pre-registered protocol. Some changes to the protocol were necessary over the course of the study. In particular, 1) planned assessments of changes in cognitive performance could not be carried out; 2) the target group of the trial was extended to include children and adolescents with experiences of violence in the family; and 3) the collection of waitlist control data was largely unsuccessful.

Clinicians (psychologists, medical doctors, social workers, nurses) with previous experience working with traumatized patients at inpatient and outpatient units, primary healthcare and asylum seeker housing units in Finland were first trained in NET. The clinicians then recruited suitable participants for the study at their corresponding units and provided NET or TAU to the participants. The therapists also acted as assessors, collecting data from the patients they were treating largely the same way they would normally track their patients’ symptoms and effects of treatment. In line with principles of pragmatic trials, the researchers were involved in the treatment practice as little as possible.

Participants were randomized into NET or TAU using sealed, opaque envelopes with a 1:1 distribution for each unit. No attempt was made to blind participants to the type of intervention they received. As measurements were based on self-reports, assessment was not blinded either. Written consent to participate in the study was requested from the participants themselves and their parents or guardians. The ethical boards of Pirkanmaa Hospital District, Tampere City Welfare Services, the Helsinki Diaconess Institute, and the Hospital District of Southwest Finland approved the study.

For participants randomized into the NET group, narrative exposure therapy was implemented as described in the 2nd edition manual (Schauer et al. 2011), with 7–10 treatment sessions of around 90 min, for a total duration of approximately three months. The TAU comparison group received whatever treatment and attention they would normally receive at the cooperating unit for a similar duration. According to information provided by the clinicians, this ranged from case management and supportive discussions to family therapy and network meetings, and non-trauma-focused psychotherapy. Changes in pharmacotherapy were not mentioned. More details on implementation, as well as the results of this trial on the effectiveness of NET vs. TAU on primary outcomes are reported elsewhere (Peltonen and Kangaslampi 2019).

### Measures

All measures were available in Finnish, English, Arabic, Dari, and Sorani (Central Kurdish) translations. For those few children or adolescents who did not speak any of these languages, an interpreter read out the questions and response alternatives to them. Assessments were carried out by the treating clinicians at the start of the intervention (T1) and at the end of the intervention (T3). For some participants, assessments were also made mid-way through the intervention (T2) and at follow-up approximately three months after the intervention (T4).

#### Posttraumatic Stress Symptoms

The Children’s Revised Impact of Events Scale (CRIES; Smith et al. 2003) is a self-report questionnaire based on DSM-IV criteria for PTSD. The children and adolescents evaluated on a 4-point scale how often they had experienced a particular symptom over the last two weeks (0 = *not at all*, 1 = *rarely*, 3 = *sometimes*, 5 = *often*). We used a total sum with a theoretical range of 0–65. In this sample, internal consistency was good at T1 (Cronbach’s α = .83, 95% CI [.75, .90]), T2 (α = .80, 95% CI [.71, .89]), T3 (α = .87, 95% CI [.81, .93]), and T4 (α = .90, 95% CI [.86, .95]).

#### Posttraumatic Cognitions

The Child Post-Traumatic Cognitions Inventory (CPTCI; Meiser-Stedman et al. 2009) is a self-report questionnaire of 25 items assessing maladaptive posttraumatic thoughts and appraisals among children and adolescents. The children and adolescents evaluated on a 4-point scale (1 = *don’t agree at all*, 2 = *don’t agree a bit*, 3 = *agree a bit*, 4 = *agree a lot*) to what extent they agreed with each statement provided. We used a total sum variable, with a theoretical range of 25–100. Internal consistency was excellent at T1 (α = .91, 95% CI [.88, .95]), T2 (α = .94, 95% CI [.91, .97]), T3 (α = .96, 95% CI [.95, .98]), and T4 (α = .97 95% CI [.95, .98]).

#### Quality of Traumatic Memory

The Trauma Memory Quality Questionnaire (TMQQ; Meiser-Stedman et al. 2007) is an 11-item self-report questionnaire on the problematic qualities of traumatic memories. An example item is “My memories of the frightening event are mostly pictures or images”. The children and adolescents evaluated on a 4-point scale (1 = *don’t agree at all*, 2 = *don’t agree a bit*, 3 = *agree a bit*, 4 = *completely agree*) how well the statements fit their traumatic memories. We used a total sum variable with a theoretical range of 11–44. Internal consistency was good to excellent at T1 (α = .88, 95% CI [.82, .93]), T2 (α = .90, 95% CI [.86, .95]), T3 (α = .90, 95% CI [.85, .94]), and T4 (α = .90, 95% CI [.86, .95]).

### Data Analysis

A large share of data was missing, a total of 28.3% at T1, 57.0% at T2, and 25.0% at T3. Due to the majority of data missing for mid-intervention measurements, we only examined pre-post changes in most of our analyses, and used T2 measurements as additional data points for repeated measures analyses of variance only. Follow-up measurements were missing for a large share of participants in a non-random manner, and we could thus not use them in our analyses.

To account for missing data, we used multiple imputation by chained equations, employing the *mice 2.9* R package (van Buuren and Groothuis-Oudshoorn 2011) to generate 50 imputed data sets to replace missing data at the item level. According to simulation studies, item-level imputation provides greater statistical power than imputing at scale level, which has been common in the past (Gottschall et al. 2012). For inclusion as a predictor for each variable with missing values, we set a .30 minimum correlation threshold. As additional predictor variables for imputation, we used available demographic variables, additional data collected on depressive symptoms (Depression Self-Rating Scale for Children; Birleson et al. 1987) and on strengths and difficulties (Strengths and Difficulties Questionnaire; Goodman 1997), as well as CRIES, CPTCI and TMQQ scores at follow-up, where available. All the following analyses are based on pooled estimates from the multiply imputed data sets and marked with an “MI” subscript.

We used repeated-measures analyses of variance to assess changes in posttraumatic cognitions and traumatic memories over the course of treatment, and differences between the treatment conditions in such changes. We further assessed the relationship between pre-post changes in posttraumatic cognitions and changes in traumatic memories with pre-post improvements in PTSS by correlation analyses.

For mediation analyses, we used maximum-likelihood path analysis implemented with the *lavaan 0.6–3* R package (Rosseel 2012), together with the *semTools 0.5–1* package (Jorgensen et al. 2018) to allow for analyses in multiply imputed datasets. We specified separate path models for CPTCI and TMQQ scores as mechanisms. In each model, CRIES scores at T3 were regressed on T1 CRIES scores, a dummy variable for type of intervention and, and CPTCI/TMQQ scores at T3 (the *b* path for mediation analysis). The CPTCI/TMQQ score at T3 was in turn regressed on CPTCI/TMQQ scores at T1, CRIES scores at T1, and the intervention dummy variable (the *a* path for mediation analysis). Because of the complexity of combining multiple imputation with bootstrapping approaches, we used the conservative asymptotic normal distribution method of constructing confidence intervals around the estimates of indirect effects, *a* * *b,* to assess the significance of mediated effects. We carried out all data processing and analyses using R 3.4.3 (R Core Team 2017). Input scripts are available upon request from the first author.

## Results

### Descriptive Statistics

Table 1 presents the levels of posttraumatic stress symptoms, maladaptive posttraumatic cognitions and problematic qualities of traumatic memories before and after treatment for the two intervention groups separately and in aggregate, based on multiply imputed data.

**Table 1:** Levels of maladaptive posttraumatic cognitions, problematic qualities of traumatic memories, and posttraumatic stress symptoms at pretest and posttest for multiply traumatized children and adolescents receiving two types of treatment for posttraumatic stress symptoms

	Narrative exposure therapy (*n* = 23)					Treatment as usual (*n* = 17)					Whole sample (*n* = 40)				
	Pretest		Posttest			Pretest		Posttest			Pretest		Posttest		
Measure	*M*	*SD*	*M*	*SD*	*d*_rm_	*M*	*SD*	*M*	*SD*	*d*_rm_	*M*	*SD*	*M*	*SD*	*d*_rm_
CRIES	37.84	14.41	28.38	14.85	0.65***	35.63	12.31	31.72	14.68	0.29	36.90	13.44	29.80	14.69	0.50***
CPTCI	55.89	13.91	52.01	18.12	0.22	51.40	11.62	50.79	14.61	0.04	53.99	13.02	51.49	16.52	0.16
TMQQ	28.03	7.81	25.99	7.64	0.26*	27.57	7.37	24.03	8.83	0.43	27.83	7.54	25.16	8.12	0.34*

*CRIES* Children’s Revised Impact of Event Sale, *CPTCI* Child Post-Traumatic Cognitions Inventory, *TMQQ* Traumatic Memory Quality Questionnaire. Pooled estimates based on 50 multiple imputation sets. * *p* < .05. *** *p* < .001

### Changes in Posttraumatic Cognitions and Traumatic Memories during NET and TAU

Repeated-measures ANOVA indicated a significant effect of Time on traumatic memories (*F*_MI_(11,333.56) = 4.77, *p* = .029), but no significant effect of Intervention (*F*_MI_(135,050.44) = 0.62, *p* = .431) or Time × Intervention interaction (*F*_MI_(14,315.37) = 0.84, *p* = .361). For posttraumatic cognitions, results indicated no significant effect of Time (*F*_MI_(13,726.70) = 1.45, *p* = 0.229), Intervention (*F*_MI_(157,323.45) = 0.75, *p* = .386) or Time × Intervention interaction (*F*_MI_(1, 4498.45) = 0.95, *p* = 0.330). There was also a significant effect of Time (*F*_MI_(131,663.39) = 9.78, *p* = .002) on posttraumatic stress symptoms, but no effect of Intervention (*F*_MI_(1,372,675.75) = 0.027, *p* = .870), or Time × Intervention (*F*_MI_(1, 15,347.50) = 1.69, *p* = .194).

Changes in PTSS from pretest to posttest correlated significantly with changes in posttraumatic cognitions from pretest to posttest (*r*_MI_ = .46, 95% CI [.11, .70], *p* = .011). Changes in PTSS from pretest to posttest likewise correlated significantly with changes in traumatic memories from pretest to posttest (*r*_MI_ = .36, 95% CI [.01, .63], *p* = .044).

### Mediation Analyses

Mediation analyses indicated that CPTCI scores at T3 predicted CRIES scores at T3, accounting for pretest CRIES scores (*b*_MI_ = 0.54, *SE* = 0.11, *p* < .001). However, participation in NET vs. TAU did not significantly predict CPTCI scores at T3, accounting for pretest levels (*b*_MI_ = −3.22, SE = 3.51, *p* = 0.359.), and there was no evidence of mediated effects on CRIES scores at T3 via CPTCI scores at T3 (indirect effect = −1.74, 95% CI [−5.61, 2.13]).

Similarly, for changes in traumatic memories, TMQQ scores at T3 predicted CRIES scores at T3, accounting for pretest CRIES scores (*b*_MI_ = 0.84, *SE* = 0.24, *p* = .001). However, participation in NET vs. TAU did not predict TMQQ scores at T3, accounting for pretest levels (*b*_MI_ = 1.52, *SE* = 2.13, *p* = .477), and there was again no evidence of mediated effects on CRIES at T3 via traumatic memories at T3 (indirect effect = 1.27, 95% CI [−2.44, 4.97]).

## Discussion

Understanding how successful treatment of posttraumatic stress symptoms (PTSS) takes place is crucial for further development, better targeting, and effective dissemination of treatment approaches. Existing evidence on mechanisms of change involved in PTSS treatment among multiply traumatized children and adolescents is very limited. Here, we examined changes in maladaptive posttraumatic cognitions (PTCs) and problematic qualities of traumatic memories as potential mechanisms of change during narrative exposure therapy and treatment as usual. Previously, a number of studies have found a link between improvements in PTCs and successful treatment of PTSS among children and adolescents (Jensen et al. 2018; McLean et al. 2015; Meiser-Stedman et al. 2017; Smith et al. 2007), though studies among children or adolescents traumatized by war and in usual care environments are lacking. In contrast, despite theoretical emphasis on their importance, the role of changes in the quality of traumatic memories in treatment of PTSS has only been tentatively shown in a single study for children and adolescents with single-incident trauma (Meiser-Stedman et al. 2017).

Our results here showed that positive changes over the course of treatment in both PTCs and the quality of traumatic memories were associated with recovery from PTSS among multiply traumatized children and adolescents. Those children who experienced improvements in cognitions, such as viewing the world as less threatening and themselves as less vulnerable, and in traumatic memories, such as describing their memories as less sensory-based and incoherent and more verbally available, saw more reduction in symptoms. However, we only found evidence of overall average improvement over the course of treatment in the quality of traumatic memories, not in PTCs.

We did not find evidence that NET would affect PTCs or the quality of traumatic memories to a greater degree than treatment as usual, as currently provided by various units in the Finnish healthcare system. This was unexpected, as based on its principles of action (Schauer et al. 2011) and earlier findings with other exposure-based therapies (McLean et al. 2015), we hypothesized that NET would be more able to effect change in cognitions and especially memories than TAU, which did not, in most cases, directly address the traumatic events. While finding TAU to have near equivalent therapeutic effects to evidence-based treatments is rather common (Kazdin 2015), it remains unclear how the largely non-trauma-focused TAU in this case also resulted in improvements in the quality of traumatic memories. As the same therapists carried out NET and TAU, some spillover effects from the provided NET training are possible. Even during TAU, therapists might have, e.g., encouraged participants to verbalize and contextualize their traumatic memories, or set in motion emotional processing of the memories outside treatment sessions.

The changes we observed in both putative mechanisms were modest overall. Employing the same measures as here, Meiser-Stedman et al. (2017) reported much greater changes in PTCs and traumatic memories from pre- to posttreatment with Cognitive Therapy for PTSD in children and adolescents after single-incident trauma. Especially the fact that NET and TAU did not appear to significantly change the maladaptive trauma-affected thinking patterns of these multiply traumatized children and adolescents needs explanation. It is possible that maladaptive, overly negative PTCs resulting from long-term, repeated exposure to interpersonal trauma, especially war and conflict, are less susceptible to change than those resulting from single, anomalous incidents. In continuously dangerous environments, appraisals of the world as dangerous and unpredictable may have been adaptive and realistic for these children and adolescents, which could explain why it is difficult to alter them in short-term treatment. Although there was no longer any acute threat to life among the participating children, the effects of uncertainty and experienced losses related to close family members remained and might contribute to the persistence of maladaptive cognitive appraisals.

One study on a psychosocial group intervention among war-affected children similarly found no significant changes in PTCs (Kangaslampi et al. 2016). Outside of conditions of war, however, other studies of trauma-focused treatment that have included adolescents exposed to repeated interpersonal trauma have observed significant changes in PTCs that have contributed to symptom reduction (e.g., Jensen et al. 2018; McLean et al. 2015). Due to the limited sample size, we could not study the possible moderating effect of experiences of war versus family violence on effectiveness of treatment or change in the suggested mechanisms. Such comparisons on the importance of type of trauma experienced for treatment effects and mechanisms of change involved are sorely needed to understand whether improvements in PTSS can be achieved through similar mechanisms for children and adolescents exposed to war, as well.

## Strengths and Limitations

The real-life, multisite usual care setting of the study, despite causing significant challenges, increases our confidence in the generalizability of our results to similar healthcare systems. We consider studying multiply traumatized children and adolescents, including those with war-related trauma, another strength of the study, as previous studies on mechanisms of change among children have often concentrated on single-incident trauma. Importantly, our planned analyses of mechanisms of change and hypotheses were preregistered before any collection of data.

However, we should also point out a number of major limitations. First, this analysis relies on self-report measures. Other types of measures for maladaptive PTCs have been called for (e.g., Schnyder et al. 2015), but not, to our knowledge, presented. For traumatic memories, alternatives include behavioral measures of the frequency of intrusions. However, such measures do not capture the qualitatively problematic nature of traumatic memories as excessively sensory, fragmented, or inadequately contextualized and integrated, but rather represent occurrence of intrusive symptoms, considered here as part of PTSS. Outsider-rated indices of fragmentation or coherence based on written or oral trauma narratives have also been used. Self-reports of the quality of traumatic memory might be less prone to the effects of distress or avoidance than providing a detailed narrative (Halligan et al. 2003). On the other hand, self-reports may be criticized for being influenced by demand characteristics (Pasupathi 2007) and for the problems inherent in trauma survivors thought to suffer from memory problems or deficits providing self-reports on their memories (O'Kearney and Perrott 2006). Self-reports of memory quality may perhaps be more adequately described as measures of meta-memory or perception of memory quality (McKinnon et al. 2017).

Further, the instruments we used to assess PTCs and traumatic memories both refer to “the event”. However, our participants had experienced multiple traumatic events and NET treatment involves exposure to all or most of them, instead of a single event. Participants were instructed to think of the worst or prototypical traumatic event. Still, this may mean the instruments did not fully capture changes in memories related to other events or cognitions reflecting the participants’ general trauma-affected view of the world. Development of research instruments more appropriate for the assessment of multiply traumatized children and adolescents is a crucial future task. In the first place, such instruments should refrain from concentrating on just a single causative event. Beyond this, they should attempt to capture more fully the wide-ranging effects repeated interpersonal trauma may have on children’s appraisals and world view, especially during sensitive periods of development.

Second, despite a moderately sized sample, due to the real-life usual care setting where the data were collected, a large portion of data was missing. This meant we could only employ pretest and posttest assessments in most analyses, and statistical power was inadequate to detect smaller effects, despite the use of sophisticated methods to deal with missing data. Overall, our results here and in the primary analyses of the trial reported elsewhere (Peltonen and Kangaslampi 2019) suggest that our sample may not have been adequately powered to detect differences between NET and TAU. Further, despite our attempts to collect follow-up data three months after treatment ended, so much follow-up data was missing in a non-random pattern that it could not be included here, except as auxiliary data to support multiple imputation. We should also note that while changes to possible pharmacotherapy were not made in the NET group nor noted in the TAU group during treatment, we cannot completely rule out possible chance differences in medication use between the groups, as we did not have access to full medical records.

Third, in the original protocol for this study (Kangaslampi et al. 2015), we planned to employ the waiting period before start of treatment as a waiting list control condition. However, waiting times were shorter than expected, and we received little data on such a waiting period. Thus, we could not compare changes during NET and TAU to a waitlist condition. Such comparisons would have been valuable to elucidate whether changes in PTCs and traumatic memories acted as mechanisms of change in PTSS reduction in treatment overall as compared with no treatment. Including both active and passive control conditions in future studies is highly advisable.

## Conclusions

Demonstrating an effect on the specific mechanism(s) proposed to account for a treatment’s effectiveness would increase our confidence in that treatment. At the same time, uncovering shared, general mechanisms that differing treatment approaches, when successful, draw upon, is crucial for refinement and better targeting of these treatments (Ehlers et al. 2010).

Acknowledging the substantial limitations of the current study, we may suggest that while the multiply traumatized children and adolescents in our study maintained much of their assessments of the world as a scary, unpredictable place and of themselves as vulnerable, they were able to moderate some of the frightening, “unspeakable” aspects of their memories (sights, sounds, and sensations) related to highly adverse events, which was linked to alleviation of PTSS. Problematic qualities of traumatic memories may form an important target of treatment, at least among those children and adolescents with multiple experiences of interpersonal trauma. However, we were unable to demonstrate that such changes would be a mechanism of change specific to NET. Additional research with larger samples is required to elucidate, first, whether improvements in the quality of traumatic memories are a major mechanism of change in the treatment of PTSS overall, and second, whether NET or similar exposure-based methods may have more of their effects via this mechanism than other forms of treatment.

In line with previous research, we found that improvements in maladaptive posttraumatic cognitions were associated with recovery from posttraumatic stress symptoms. We did not, however, observe significant changes in such cognitions over the course of treatment. This may suggest effecting change in posttraumatic cognitions is more challenging for multiply traumatized children and adolescents overall or for those affected by war and conflict in particular. Children and adolescents traumatized by war have been seriously underrepresented in studies of trauma-focused treatment overall, and in studies of the mechanisms of such treatment in particular. Studying whether the psychological mechanisms that lead to symptom improvement among this population may differ from those exposed to single-incident trauma or repeated trauma not related to war is an important future task. In any case, preregistered analyses of mechanisms of change should be included in all future trials of treatments for PTSS.

## References

[CR1] Alghamdi M, Hunt N, Thomas S (2015). The effectiveness of narrative exposure therapy with traumatized firefighters in Saudi Arabia: A randomized controlled study. Behaviour Research and Therapy.

[CR2] Bedard-Gilligan M, Zoellner LA, Feeny NC (2017). Is trauma memory special? Trauma narrative fragmentation in PTSD: Effects of treatment and response. Clinical Psychological Science.

[CR3] Birleson P, Hudson I, Buchanan DG, Wolff S (1987). Clinical evaluation of a self-rating scale for depressive disorder in childhood (depression self-rating scale). Journal of Child Psychology and Psychiatry.

[CR4] Brewin CR (2014). Episodic memory, perceptual memory, and their interaction: Foundations for a theory of posttraumatic stress disorder. Psychological Bulletin.

[CR5] Brown RC, Witt A, Fegert JM, Keller F, Rassenhofer M, Plener PL (2017). Psychosocial interventions for children and adolescents after man-made and natural disasters: A meta-analysis and systematic review. Psychological Medicine.

[CR6] Catani C, Kohiladevy M, Ruf M, Schauer E, Elbert T, Neuner F (2009). Treating children traumatized by war and tsunami: A comparison between exposure therapy and meditation relaxation in North-East Sri Lanka. BMC Psychiatry.

[CR7] Cooper AA, Clifton EG, Feeny FC (2017). An empirical review of potential mediators and mechanisms of prolonged exposure therapy. Clinical Psychology Review.

[CR8] Dalgleish T (2004). Cognitive theories of posttraumatic stress disorder: The evolution of multi-representational theorizing. Psychological Bulletin.

[CR9] Desrochers AB, Beaulieu-Prévost D, Desautels J, Békés V, Belleville G, Guay S, Marchand A (2016). Gender and changes in trauma narrative following CBT for PTSD. Journal of Aggression, Maltreatment and Trauma.

[CR10] Ehlers A, Clark D (2000). A cognitive model of posttraumatic stress disorder. Behaviour Research and Therapy.

[CR11] Ehlers A, Bisson J, Clark DM, Creamer M, Pilling S, Richards D, Schnurr PP, Turner S, Yule W (2010). Do all psychological treatments really work the same in posttraumatic stress disorder?. Clinical Psychology Review.

[CR12] Ertl V, Pfeiffer A, Schauer E, Elbert T, Neuner F (2011). Community-implemented trauma therapy for former child soldiers in northern Uganda: A randomized controlled trial. JAMA.

[CR13] Foa EB, Rothbaum BA (1998). Treating the trauma of rape: Cognitive behavioral therapy for PTSD.

[CR14] Gillies D, Maiocchi L, Bhandari AP, Taylor F, Gray C, O’Brien L (2016). Psychological therapies for children and adolescents exposed to trauma. Cochrane Database Systematic Reviews.

[CR15] Goodman R (1997). The strengths and difficulties questionnaire: A research note. Journal of Child Psychology and Psychiatry.

[CR16] Gottschall AC, West SG, Enders CK (2012). A comparison of item-level and scale-level multiple imputation for questionnaire batteries. Multivariate Behavioral Research.

[CR17] Halligan SL, Michael T, Clark DM, Ehlers A (2003). Posttraumatic stress disorder following assault: The role of cognitive processing, trauma memory, and appraisals. Journal of Consulting and Clinical Psychology.

[CR18] Jensen TK, Holt T, Ormhaug SM, Fjermestad KW, Wezel-Larsen T (2018). Change in post-traumatic cognitions mediates treatment effects for traumatized youth – A randomized controlled trial. Journal of Counseling Psychology.

[CR19] Johansson P, Høglend P (2007). Identifying mechanisms of change in psychotherapy: Mediators of treatment outcome. Clinical Psychology and Psychotherapy.

[CR20] Jorgensen, T. D., Pornprasertmanit, S., Schoemann, A. M., & Rosseel, Y. (2018). semTools: Useful tools for structural equation modeling. R package version 0.5–1. Available at https://CRAN.R-project.org/package=semTools. Accessed 22 Jan 2019.

[CR21] Kangaslampi S, Garoff F, Peltonen K (2015). Narrative exposure therapy for immigrant children traumatized by war: Study protocol for a randomized controlled trial of effectiveness and mechanisms of change. BMC Psychiatry.

[CR22] Kangaslampi S, Punamäki R-L, Qouta S, Diab M, Peltonen K (2016). Psychosocial group intervention among war-affected children: An analysis of changes in posttraumatic cognitions. Journal of Traumatic Stress.

[CR23] Kazdin AE (2007). Mediators and mechanisms of change in psychotherapy research. Annual Review of Clinical Psychology.

[CR24] Kazdin A (2015). Treatment as usual and routine care in research and clinical practice. Clinical Psychology Review.

[CR25] Kleim B, Grey N, Wild J, Nussbeck FW, Stott R, Hackmann A, Clark DM, Ehlers A (2013). Cognitive change predicts symptom reduction with cognitive therapy for posttraumatic stress disorder. Journal of Consulting and Clinical Psychology.

[CR26] Kumpula MJ, Pentel KZ, Foa EB, LeBlanc NJ, Bui E, McSweeney LB, Knowles K, Bosley H, Simon NM, Rauch S (2017). Temporal sequencing of change in posttraumatic cognitions and PTSD symptom reduction during prolonged exposure therapy. Behavior Therapy.

[CR27] McKinnon A, Brewer N, Meiser-Stedman R, Nixon RDV (2017). Trauma memory characteristics and the development of acute stress disorder and post-traumatic stress disorder in youth. Journal of Behavior Therapy and Experimental Psychiatry.

[CR28] McLean CP, Yeh R, Rosenfield D, Foa EB (2015). Changes in negative cognitions mediate PTSD symptom reductions during client-centered therapy and prolonged exposure for adolescents. Behaviour Research and Therapy.

[CR29] Meiser-Stedman R, Smith P, Yule W, Dalgleish T (2007). The trauma memory quality questionnaire: Preliminary development and validation of a measure of trauma memory characteristics for children and adolescents. Memory.

[CR30] Meiser-Stedman R, Smith P, Bryant R, Salmon K, Yule W, Dalgleish T, Nixon R (2009). Development and validation of the child post-traumatic cognitions inventory (CPTCI). Journal of Child Psychology and Psychiatry.

[CR31] Meiser-Stedman R, Smith P, McKinnon A, Dixon C, Trickey D, Ehlers A, Clark DM, Boyle A, Watson P, Goodyer I, Dalgleish T (2017). Cognitive therapy as an early treatment for post-traumatic stress disorder in children and adolescents: A randomized controlled trial addressing preliminary efficacy and mechanisms of action. Journal of Child Psychology and Psychiatry.

[CR32] Mørkved N, Hartmann K, Aarsheim L, Holen D, Milde A, Bomyea J, Thorp S (2014). A comparison of narrative exposure therapy and prolonged exposure therapy for PTSD. Clinical Psychology Review..

[CR33] O'Kearney R, Perrott K (2006). Trauma narratives in posttraumatic stress disorder: A review. Journal of Traumatic Stress.

[CR34] O'Kearney R, Speyer J, Kenardy J (2007). Children's narrative memory for accidents and their posttraumatic distress. Applied Cognitive Psychology.

[CR35] Pabst A, Schauer M, Bernhardt K, Ruf-Leuschner M, Goder R, Elbert T (2014). Evaluation of narrative exposure therapy (NET) for borderline personality disorder with comorbid posttraumatic stress disorder. Clinical Neuropsychiatry.

[CR36] Pasupathi M (2007). Telling and the remembered self: Linguistic differences in memories for previously disclosed and previously undisclosed events. Memory.

[CR37] Peltonen K, Kangaslampi S (2019). Treating children and adolescents with multiple traumas: A randomized clinical trial of narrative exposure therapy. European Journal of Psychotraumatology.

[CR38] R Core Team (2017). R: A language and environment for statistical computing. R Foundation for Statistical Computing, Vienna, Austria. Available at https://www.R-project.org/. Accessed 22 Jan 2019.

[CR39] Robjant K, Fazel M (2010). The emerging evidence for narrative exposure therapy: A review. Clinical Psychology Review.

[CR40] Rosseel Y (2012). Lavaan: An R package for structural equation modeling. Journal of Statistical Software.

[CR41] Rubin DC, Deffler SA, Ogle CM, Dowell NM, Graesser AC, Beckham JC (2016). Participant, rater, and computer measures of coherence in posttraumatic stress disorder. Journal of Abnormal Psychology.

[CR42] Ruf M, Schauer M, Neuner F, Catani C, Schauer E, Elbert T (2010). Narrative exposure therapy for 7- to 16-year-olds: A randomized controlled trial with traumatized refugee children. Journal of Traumatic Stress.

[CR43] Salmond C, Meiser-Stedman R, Glucksman E, Thompson P, Dalgleish T, Smith P (2011). The nature of trauma memories in acute stress disorder in children and adolescents. Journal of Child Psychology and Psychiatry.

[CR44] Schaal S, Elbert T, Neuner F (2009). Narrative exposure therapy versus interpersonal psychotherapy: A pilot randomized controlled trial with Rwandan genocide orphans. Psychotherapy and Psychosomatics.

[CR45] Schauer M, Neuner F, Elbert T (2011). *Narrative Exposure Therapy: A short-term treatment for traumatic stress disorders*.

[CR46] Schnyder U, Ehlers A, Elbert T, Foa EB, Gersons BPR, Resick PA (2015). Psychotherapies for PTSD: What do they have in common?. European Journal of Psychotraumatology.

[CR47] Smith P, Perrin S, Dyregrov A, Yule W (2003). Principal components analysis of the impact of event scale with children in war. Personality and Individual Differences.

[CR48] Smith P, Yule W, Perrin S, Tranah T, Dalgleish T, Clark DM (2007). Cognitive-behavioral therapy for PTSD in children and adolescents: A preliminary randomized controlled trial. JAMA Child and Adolescent Psychiatry.

[CR49] Sripada RK, Rauch SAM, Liberzon I (2016). Psychological mechanisms of PTSD and its treatment. Current Psychiatry Reports.

[CR50] van Buuren S, Groothuis-Oudshoorn K (2011). Mice: Multivariate imputation by chained equations in R. Journal of Statistical Software.

[CR51] Zalta AK (2015). Psychological mechanisms of effective cognitive–behavioral treatments for PTSD. Current Psychiatry Reports.

[CR53] Zang Y, Hunt N, Cox T (2013). A randomised controlled pilot study: The effectiveness of narrative exposure therapy with adult survivors of the Sichuan earthquake. BMC Psychiatry.

